# Mandatory lane-changing decision and control method based on game theory

**DOI:** 10.1371/journal.pone.0350209

**Published:** 2026-06-12

**Authors:** Zhe Wang, Xinyi Zhang, Haoze Ren, Liu Yang

**Affiliations:** 1 College of Vehicle and Transportation Engineering, Taiyuan University of Science and Technology, Taiyuan, China; 2 Guizhou Jonyang Kinetics Co., Ltd., Guiyang, China; Nanjing Forestry University, CHINA

## Abstract

This paper proposes a lane-changing decision and control framework for mandatory lane-changing scenarios based on stage game decision-making. The lane-changing process is discretized, and payoff functions are constructed for the autonomous vehicle and the following vehicles in the target lane, enabling adaptive adjustment of driving strategies and planned trajectories according to surrounding vehicle interactions. The optimal lane-changing decision is dynamically updated by incorporating environmental information and interactive feedback from surrounding vehicles. For trajectory tracking, a composite error combining lateral displacement and heading angle deviations is defined and constrained using a preset performance boundary. The constrained error is transformed into an equivalent unconstrained form, and a sliding mode controller is designed to ensure robust tracking performance. A joint simulation platform integrating traffic simulation and driver-in-the-loop experiments is established to evaluate the proposed framework. A preliminary study involving three human drivers with different driving tendencies is conducted to analyze interaction behaviors in mandatory lane-changing scenarios. The experimental results provide initial insights into the effectiveness of the proposed approach under representative conditions.

## 1 Introduction

Autonomous vehicles have attracted increasing attention as a promising solution to alleviate traffic congestion and improve road safety in future transportation systems [1–3]. Among the core enabling technologies, the ability to perform lane-changing maneuvers in a reasonable, safe, and efficient manner remains a critical challenge and has become a central research topic worldwide.

Lane-changing behavior can generally be classified into discretionary lane changing and mandatory lane changing according to the driving scenario [4,5]. Discretionary lane changing refers to voluntary maneuvers initiated by drivers to achieve higher speeds or greater driving comfort, even when such maneuvers are not strictly required. In contrast, mandatory lane changing arises from external constraints, such as lane drops, obstacles, or required turning movements at downstream intersections. In both cases, lane-changing maneuvers inevitably impose disturbances on adjacent traffic streams, particularly by forcing following vehicles in the target lane to decelerate. These disturbances can degrade traffic efficiency and stability, leading to adverse phenomena such as traffic breakdown, capacity reduction [6], stop-and-go oscillations [7], and increased safety risks. Compared with discretionary lane changing, mandatory lane-changing maneuvers typically exert a more pronounced negative impact on traffic flow and safety due to their frequent occurrence and coercive nature [8]. Consequently, this study focuses on the modeling and control of mandatory lane-changing behavior.

Most existing studies on lane-changing decision-making model autonomous vehicles as isolated agents, treating surrounding vehicles merely as moving obstacles. While such approaches simplify the problem formulation, they fail to capture the inherently interactive and dynamic nature of lane-changing behavior in traffic flow. In reality, surrounding vehicles continuously provide feedback that influences the lane-changing vehicle’s decisions and trajectory planning. Moreover, deviations between the planned and actual trajectories further affect subsequent driving decisions, forming a closed-loop interaction process.

To address the limitations of existing lane-changing methods, this paper proposes a unified lane-changing decision and trajectory planning framework based on stage game theory, with several key innovations. First, unlike conventional game-theoretic approaches that typically consider single-stage interactions or static decision processes, the proposed framework models the lane-changing process as a multi-stage dynamic game. This enables the autonomous vehicle to continuously update its strategy by incorporating real-time interaction feedback from surrounding vehicles. Second, a tailored payoff function is designed to explicitly capture the trade-offs among safety, efficiency, and comfort, providing a more interpretable and application-oriented decision-making mechanism. Third, the proposed framework integrates decision-making and trajectory generation in a unified manner, allowing both to be adaptively adjusted in response to evolving traffic conditions. As a result, the method enhances interaction awareness and robustness compared with existing game-theoretic lane-changing approaches.

In terms of trajectory tracking control, this paper further introduces a preset performance–based control framework to address the limitations of existing methods, which predominantly focus on steady-state accuracy while neglecting transient safety. The key innovation lies in explicitly constraining the transient tracking performance to ensure safety during highly interactive maneuvers. Specifically, a composite tracking error that integrates lateral displacement and heading angle deviations is defined and constrained within a prescribed performance boundary. This formulation ensures that tracking errors remain within safety-critical limits throughout the maneuver. The constrained error dynamics are then transformed into an equivalent unconstrained form, upon which a sliding mode controller is designed to guarantee robust and smooth tracking performance. Compared with conventional control strategies, the proposed method provides improved transient response and stronger safety guarantees, which are essential for lane-changing scenarios under dynamic interactions.

The remainder of this paper is organized as follows. Section 2 reviews major modeling approaches and game-theoretic methods for mandatory lane-changing behavior. Section 3 presents the proposed lane-changing decision and control framework. Section 4 reports comparative simulations and experimental results. Finally, conclusions are drawn in Section 5.

## 2 Literature review

### 2.1 Lane-changing decision-making models

Given the critical role of lane-changing decision-making in autonomous driving systems, a substantial body of research has been devoted to modeling and understanding lane-changing behavior. Existing approaches can be broadly categorized into rule-based, data-driven, and game-theoretic paradigms, each offering distinct advantages and limitations in terms of interpretability, adaptability, and interaction modeling.

Early studies primarily relied on rule-based formulations, where lane-changing decisions are governed by predefined logical conditions. Representative work by Gipps [9] established a foundational framework for modeling lane-selection priorities in multilane traffic environments, while subsequent studies further decomposed the lane-changing process into multiple stages, including motivation generation, target lane selection, gap acceptance, and execution [10]. With the increasing availability of real-world trajectory datasets, such as NGSIM, later works incorporated empirical validation into rule-based models [11] and enhanced decision-making by integrating safety and efficiency considerations through artificial potential field formulations [12]. Despite their computational efficiency and interpretability, these approaches generally treat surrounding vehicles as passive entities, limiting their ability to capture interactive and dynamic traffic behaviors.

In contrast, data-driven approaches, particularly those based on neural networks, have demonstrated strong capability in capturing complex and nonlinear lane-changing behaviors directly from data. Early studies applied neural networks to predict lane-changing decisions in simplified scenarios [13], while subsequent work improved prediction accuracy by incorporating asymmetric lane-changing characteristics and traffic features [14]. More recent advances employ deep learning architectures, such as recurrent neural networks combined with driver behavior models, to better capture temporal dependencies and contextual interactions [15]. However, these approaches are primarily designed for behavior prediction rather than decision-making and control, and their black-box nature limits interpretability in safety-critical applications.

Rule-based approaches are among the earliest methods for modeling lane-changing behavior. These methods typically rely on predefined rules or heuristics derived from traffic regulations or empirical driving data. For example, gap acceptance models and safety distance rules are commonly used to determine whether a lane change is feasible. Although rule-based methods are computationally efficient and easy to implement, they often lack flexibility and struggle to capture complex interactions among multiple vehicles in dynamic traffic environments [8,16].

Optimization-based approaches formulate lane-changing as an optimal control or trajectory planning problem, where an objective function—such as minimizing travel time, energy consumption, or tracking error—is optimized subject to vehicle dynamics and safety constraints. These methods can generate smooth and dynamically feasible trajectories and have been widely applied in autonomous driving systems. However, most optimization-based approaches assume passive surrounding vehicles and therefore have limited capability in explicitly modeling interactive behaviors among multiple agents [17].

To explicitly account for interactions among vehicles, game-theoretic approaches have gained increasing attention. By modeling vehicles as rational agents engaged in strategic interactions, these methods provide a principled framework for capturing competitive and cooperative behaviors in traffic systems. Building upon classical equilibrium concepts, such as Wardrop equilibrium [18] and its relation to Nash equilibrium, game-theoretic models have been developed to describe lane-changing behavior under interactive environments. In mandatory lane-changing scenarios, where conflicts between vehicles are inevitable, these models are particularly effective in representing negotiation processes between lane-changing and surrounding vehicles. For instance, non-cooperative game models have been proposed to capture driver perception, judgment, and behavioral characteristics [19], while subsequent studies incorporated connected vehicle information into payoff functions to enable dynamic decision-making [20]. In addition, recent research has explored integrated frameworks that jointly optimize lane-changing decisions and motion planning within a prediction horizon [21], as well as models that explicitly incorporate conflict risks and roadway factors into utility design. Furthermore, earlier studies have also investigated the characteristics of mandatory lane-changing behavior using game-theoretic formulations, emphasizing the role of conflict time in utility design [22].

Despite these advances, several challenges remain. Existing rule-based and data-driven approaches often lack explicit modeling of vehicle interactions, while many game-theoretic models primarily focus on decision-level analysis without tightly integrating trajectory planning and control. Moreover, the coupling between decision-making and control under dynamic and interactive environments remains insufficiently addressed. These limitations motivate the development of unified frameworks capable of simultaneously achieving interaction-aware decision-making and robust trajectory execution in complex mandatory lane-changing scenarios.

### 2.2 Trajectory tracking control

In the field of vehicle trajectory tracking control, sliding mode control (SMC) has been widely adopted due to its structural simplicity, inherent robustness to model uncertainties, and ease of implementation. By explicitly coupling lateral displacement and heading angle errors in Cartesian coordinates, SMC-based approaches are capable of achieving high-precision trajectory tracking performance. Recent studies have further enhanced the robustness of SMC frameworks through the integration of higher-order sliding mode techniques and disturbance observers, which effectively attenuate the influence of external disturbances and measurement noise under uncertain operating conditions [23,24].

Despite these advantages, conventional SMC schemes are prone to chattering phenomena, which may degrade control accuracy and adversely affect ride comfort. To address this limitation, a range of improved SMC strategies have been developed from different perspectives. These efforts include redesigning sliding surfaces and incorporating state estimation mechanisms to improve control smoothness and reduce chattering effects [25], as well as integrating SMC with advanced control frameworks to enhance multi-objective tracking capability and convergence performance [26]. In addition, terminal sliding mode formulations have been explored to achieve finite-time convergence while jointly considering lateral and heading tracking errors, demonstrating improved tracking accuracy and robustness [27].

Overall, existing studies have significantly advanced the performance of SMC-based trajectory tracking control in terms of robustness and accuracy. However, most approaches primarily focus on controller design and performance improvement, while the integration of SMC with higher-level decision-making and interaction-aware control strategies remains insufficiently explored, particularly in complex and dynamic traffic environments.

### 2.3 Motivation and contribution of this paper

Most existing studies on lane-changing decision-making model autonomous vehicles as isolated agents, treating surrounding vehicles as passive moving obstacles and thereby neglecting the intrinsic characteristics of traffic flow and the dynamic interactions among vehicles. However, lane-changing is inherently an interactive and adaptive process, where surrounding vehicles continuously respond to the maneuver of the subject vehicle. Such interactions not only influence the feasibility and safety of the planned trajectory, but also induce deviations between the desired and actual vehicle states, which in turn affect subsequent decision-making and control actions. The lack of an integrated framework that simultaneously captures interaction-aware decision-making and trajectory evolution limits the applicability of existing approaches in complex and dynamic traffic environments.

To address this limitation, this paper proposes a unified lane-changing decision and trajectory planning framework based on stage game theory. Specifically, payoff functions are constructed to characterize the strategic interactions between the autonomous vehicle and the following vehicle in the target lane. By discretizing the lane-changing process into multiple decision stages, the proposed framework enables the autonomous vehicle to dynamically update its driving strategy by jointly considering the surrounding traffic conditions and the interactive feedback from competing vehicles. As a result, both the lane-changing decision and the desired trajectory can be adaptively adjusted in real time, enhancing the interaction awareness and robustness of the planning process.

In terms of trajectory tracking control, existing methods predominantly focus on steady-state tracking accuracy, with insufficient attention to transient performance. However, during lane-changing maneuvers, the intensified interactions among vehicles significantly elevate collision risks, making large transient tracking errors unacceptable from a safety-critical perspective. To this end, a preset performance–based control framework is developed to explicitly regulate both transient and steady-state behaviors. A composite tracking error, integrating lateral displacement and heading angle deviations, is defined and constrained within a prescribed performance boundary. The constrained error dynamics are subsequently transformed into an equivalent unconstrained form, upon which a sliding mode controller is designed to ensure robust, smooth, and safety-critical trajectory tracking throughout the entire lane-changing process.

## 3 Methods

This paper investigates decision-making and control strategies for mandatory lane-changing scenarios, with particular emphasis on operations in the vicinity of signalized intersections. The proposed lane-changing framework for autonomous vehicles consists of two coupled modules: a lane-change decision module and a trajectory tracking control module. The interaction between the lane-changing vehicle and surrounding traffic during the maneuver is modeled as a Stackelberg leader–follower game, where the autonomous vehicle acts as the leader and dynamically adjusts its lane-changing decisions according to prevailing traffic conditions.

When the competing vehicle in the target lane (denoted as LV) exhibits low aggressiveness and the surrounding conditions are favorable, the autonomous vehicle proceeds with the lane-changing maneuver to maximize its overall benefit. Conversely, if the LV demonstrates aggressive driving behavior, continued lane-changing would increase collision risk; under such circumstances, the autonomous vehicle suspends the maneuver by maintaining its longitudinal position and waits for a more favorable opportunity. Moreover, when the lateral distance between the autonomous vehicle and the LV falls below a safety threshold, the vehicle retreats toward the centerline of the current lane to further mitigate collision risk. The vehicle’s urgency to execute the lane change is also influenced by its distance to the guide lane, with closer proximity indicating a higher necessity to complete the maneuver.

### 3.1 The vicinity of signalized intersections

As a critical node in urban road networks, the operational efficiency of signalized intersections has a significant influence on travelers’ experience and overall traffic performance. Owing to the high concentration of vehicle conflicts and the complexity of control and decision-making in the vicinity of signalized intersections, these areas are particularly prone to reduced traffic efficiency and elevated safety risks [28].

In practice, such issues are not solely determined by the geometric and control characteristics of signalized intersections, but are also closely related to heterogeneity in drivers’ driving styles and operational objectives [29,30]. When vehicles approach the guide lane, drivers must simultaneously account for signal phase transitions and the motion states of adjacent vehicles. Inadequate handling of these competing demands may lead to indecision between proceeding and stopping, a phenomenon commonly referred to as the dilemma zone problem. To address this challenge, recent studies have extended the research scope beyond the guide lane itself and defined the guide lane together with its adjacent upstream and downstream regions as the vicinity of signalized intersections (VSI) [31,32].

**Fig 1:**
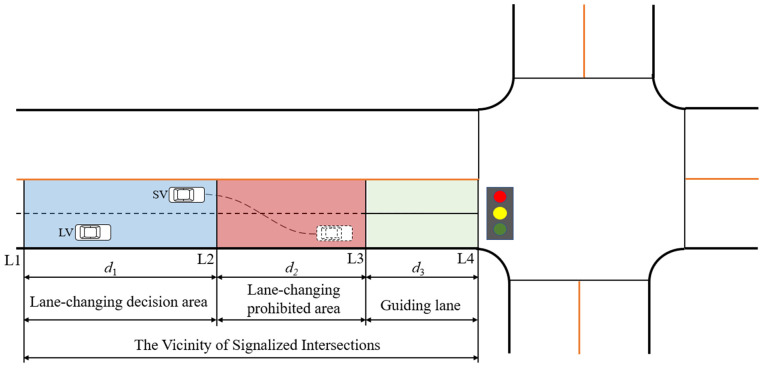
Vehicle lane change in the vicinity of traffic signals.

In the VSI scenario illustrated in Fig 1, an autonomous vehicle is required to execute a right-turn maneuver at the intersection. To complete this task, the vehicle must first perform a lane change from its initial lane to the target lane. However, this lane-changing maneuver is subject to spatial constraints imposed by the guide lane. Specifically, the subject vehicle (SV) must complete the lane change before reaching position L3 in order to satisfy the intended driving task; otherwise, the vehicle must either stop and wait or continue straight toward the next intersection. To accurately characterize lane-changing behavior in the VSI, the control region is partitioned into three subareas: a lane-change execution area, a lane-change prohibition area, and the guide lane. The lane-change execution area specifies the region within which the vehicle is required to initiate the lane-changing maneuver, whereas the lane-change prohibition area denotes regions where lane-changing conditions are no longer satisfied and the maneuver must be abandoned. It should be noted that, according to the dynamic trajectory planning framework introduced in Section III, the boundaries of these regions are not fixed but vary dynamically with the vehicle’s speed and position.

### 3.2 Lane-changing decision at cyber layer and trajectory tracking control model at physical layer

When the subject vehicle (SV) is required to execute a right-turn maneuver at the current intersection, it must first perform a lane change to reach the target lane. However, this maneuver inevitably encroaches on the right-of-way of the following vehicle in the target lane, thereby disturbing its normal driving behavior and inducing a reactive response. As a result, the lane-changing process gives rise to pronounced conflicts of interest between the two vehicles.

From a decision-making perspective, this interaction reflects a trade-off among safety, efficiency, and comfort. Specifically, safety is associated with maintaining sufficient inter-vehicle distance and avoiding collision risk, efficiency relates to completing the lane change within a limited time window to meet route requirements, and comfort is reflected in smooth acceleration and deceleration profiles. These competing objectives are inherently coupled, as aggressive maneuvers may improve efficiency but compromise safety and comfort, while overly conservative actions may ensure safety at the expense of efficiency.

Accordingly, this paper focuses on modeling the interactive game relationship between the SV and the following vehicle, where the payoff function is designed to explicitly capture the balance among safety, efficiency, and comfort under dynamic interaction conditions.

**Fig 2:**
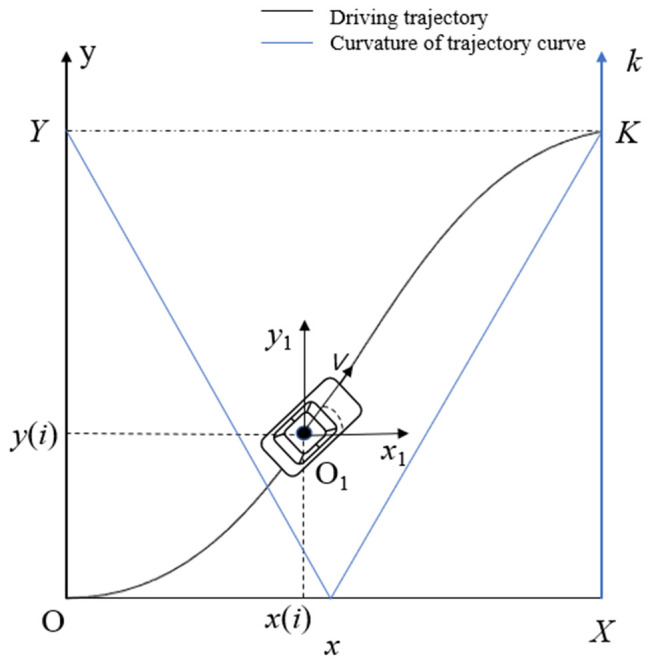
Cubic polynomial trajectory.

As illustrated in Fig 2, the subject vehicle SV performs a lane change from Lane 1 to Lane 2. At a given time instant, the lateral position of SV is depicted in Fig 2. Under this configuration, the strategy set of the subject vehicle SV is denoted by $\Gamma_{1}$, while the strategy set of the following vehicle in the target lane, denoted as LV, is represented by $\Gamma_{2}$, as defined below.


$$\begin{array}{c} \Gamma_{1}=\{\text{lane change},\text{ drive straight},\text{ rollback}\}, \end{array}$$

$$\begin{array}{c} \Gamma_{2}=\{\text{acceleration},\text{ deceleration}\}. \end{array}$$

Then, the game payoff matrix of vehicles SV and LV is obtained as shown in Table 1, where $U_{SV}(i,j)\;(i=1,2;\;j=1,2,3)$ denotes the payoff of vehicle SV when SV selects strategy *S*_*j*_ and vehicle LV selects strategy *L*_*i*_, and *U*_LV_(*i*, *j*) represents the payoff of vehicle LV under the same strategy combination.

**Table 1:** Payoff matrix.

		SV		
		*S*_1_	*S*_2_	*S*_3_
LV	*L*_1_	$\begin{array}{c} U_{LV}(1,1)\\ U_{SV}(1,1) \end{array}$	$\begin{array}{c} U_{LV}(1,2)\\ U_{SV}(1,2) \end{array}$	$\begin{array}{c} U_{LV}(1,3)\\ U_{SV}(1,3) \end{array}$
	*L*_2_	$\begin{array}{c} U_{LV}(2,1)\\ U_{SV}(2,1) \end{array}$	$\begin{array}{c} U_{LV}(2,2)\\ U_{SV}(2,2) \end{array}$	$\begin{array}{c} U_{LV}(2,3)\\ U_{SV}(2,3) \end{array}$

Furthermore, in the vicinity of signalized intersections, the primary objective of the vehicle’s lane-changing maneuver is to ensure successful completion of the intended route. Failure to complete the lane change within the available control region incurs a substantially higher cost to the vehicle. Accordingly, these considerations are incorporated into the formulation of the payoff function for the lane-changing vehicle, which is defined as follows:


$$\begin{array}{c} U_{SV}=(1-\beta_{SV})\;U_{sa1}+\beta_{SV}\;U_{sp1} \end{array}$$ (1)

where $\beta_{SV}$ denotes the weighting coefficient characterizing the driving style of vehicle SV, with larger values indicating more aggressive driving behavior. *U*_*sa*1_ represents the safety benefit of vehicle SV, while *U*_*sp*1_ denotes the space benefit of vehicle SV. The corresponding formulations of *U*_*sa*1_ and *U*_*sp*1_ are given as follows:


$$\begin{array}{c} U_{sa1}=\left\{ \begin{array}{cc} 1, & d>W+a,\\ u_{1}\;\left(1+\frac{W-d}{a}\right)+\frac{d-W}{a}, & W<d\leq W+a,\\ u_{1}, & d\leq W, \end{array}\right. \end{array}$$ (2)

$$\begin{array}{c} U_{sp1}=\left\{ \begin{array}{cc} -1, & T_{h}<0,\\ \frac{2}{3}\;T_{h}-1, & 0\leq T_{h}<3,\\ 1, & T_{h}\geq 3, \end{array}\right. \end{array}$$ (3)

where *d* denotes the lateral distance between the SV and the LV, *W* represents the vehicle width, *a* is the predefined safe lateral distance between the two vehicles, and *u*_1_ denotes the safety factor associated with vehicle SV, which is computed as follows:


$$\begin{array}{c} u_{1}=\left\{ \begin{array}{cc} 1, & T_{h}\leq -T_{e1},\\ \frac{2|T_{h}|}{T_{e1}}-1, & -T_{e1}<T_{h}\leq 0,\\ \min\;\left(\frac{2T_{h}}{T_{e1}}-1,\;1\right), & T_{h}>0, \end{array}\right. \end{array}$$ (4)

where *T*_*h*_ denotes the headway between the SV and LV, *T*_*e*1_ represents the expected headway of vehicle SV, which is defined as $T_{e1}=\min(3,\;T_{1}),$ where *T*_1_ denotes the initial headway between the FV and the SV. The corresponding formulations of *T*_*h*_ and *T*_1_ are given as follows:


$$\begin{array}{c} T_{h}=\left\{ \begin{array}{cc} \frac{P_{SV}-P_{LV}}{v_{SV}}, & P_{SV}<P_{LV},\\ \frac{P_{SV}-P_{LV}}{v_{LV}}, & P_{SV}\geq P_{LV}, \end{array}\right. \end{array}$$ (5)

$$\begin{array}{c} T_{1}=\frac{P_{FV}-P_{SV}}{v_{SV}}, \end{array}$$ (6)

where *P*_SV_ and *P*_LV_ denote the longitudinal positions of the subject vehicle (SV) and the following vehicle (LV), respectively, and *v*_SV_ and *v*_LV_ represent their corresponding longitudinal velocities.

As the vehicle approaches the lane-change prohibition region, its urgency to complete the lane-changing maneuver increases. To successfully accomplish the lane-change task under increasingly restrictive spatial conditions, the driving style of the lane-changing vehicle is required to become more aggressive. Even under extreme conditions, as long as the safety constraints are satisfied, the lane-change maneuver should be executed to fulfill the task requirements. Accordingly, the driving style coefficient of the lane-changing vehicle is defined as follows:


$$\begin{array}{c} \beta_{SV}=\max\;\left(\frac{10}{\sigma_{2}\sqrt{2\pi }}\exp\;\left(-\frac{d_{x}^{2}}{2\sigma_{2}^{2}}\right),\;\beta \right),\;d_{x}\in [0,d_{2}], \end{array}$$ (7)

where β denotes the default driving style coefficient, $\sigma_{2}$ is a positive constant, and *d*_*x*_ represents the longitudinal distance between the current position of vehicle SV and position L2. When the subject vehicle SV performs a lane-changing maneuver, it inevitably encroaches on the driving space of the following vehicle LV and may introduce potential collision risks. Consequently, driving space and safety are the primary concerns for vehicle LV. The payoff function of vehicle LV is therefore defined as [33]:


$$\begin{array}{c} U_{LV}=(1-\beta_{LV})\;U_{sa2}+\beta_{LV}\;U_{sp2}, \end{array}$$ (8)

where $\beta_{LV}$ denotes the weighting coefficient characterizing the driving style of the LV driver, with larger values corresponding to more aggressive driving behavior.*U*_*sa*2_ denotes the safety benefit of the following vehicle (LV), and *U*_*sp*2_ represents the space benefit of vehicle LV. The corresponding formulations of *U*_*sa*2_ and *U*_*sp*2_ are defined as follows:


$$\begin{array}{c} U_{sa2}=\left\{ \begin{array}{cc} 1, & d>w+a,\\ u_{2}\;\left(1+\frac{w-d}{a}\right)+\frac{d-w}{a}, & w<d\leq w+a,\\ u_{2}, & d\leq w, \end{array}\right. \end{array}$$ (9)

$$\begin{array}{c} U_{sp2}=\left\{ \begin{array}{cc} 1, & T_{h}<0,\\ 1-\frac{2}{3}T_{h}, & 0\leq T_{h}<3,\\ -1, & T_{h}\geq 3, \end{array}\right. \end{array}$$ (10)

where *d* denotes the lateral distance between the subject vehicle (SV) and the following vehicle (LV), *w* represents the vehicle width, *a* is the predefined safe lateral distance between the two vehicles, and *T*_*h*_ denotes the headway of vehicle SV relative to vehicle LV. The parameter *u*_2_ represents the safety factor associated with vehicle LV, which is computed as follows:


$$\begin{array}{c} u_{2}=\left\{ \begin{array}{cc} 1, & T_{h}\leq -T_{e2},\\ \frac{2|T_{h}|}{T_{e2}}-1, & -T_{e2}<T_{h}\leq T_{e2},\\ 1, & T_{h}>T_{e2}, \end{array}\right. \end{array}$$ (11)

where *T*_*e*2_ denotes the expected headway of vehicle LV, defined as $T_{e2}=\min(3,\;T_{2})$. In solving the game model, each participant selects a strategy to optimize its respective objective. LV in the target lane, the Stackelberg equilibrium is employed. The resulting optimal strategies are given by


$$\begin{array}{c} \left\{ \begin{array}{c} \xi_{SV}^{*}=\text{arg}\max_{\xi_{SV}\in \Gamma_{1}}U_{SV}(\xi_{SV},\xi_{LV}^{*}(\xi_{SV})),\\ \xi_{LV}^{*}(\xi_{SV})=\text{arg}\max_{\xi_{LV}\in \Gamma_{2}}U_{LV}(\xi_{SV},\xi_{LV}), \end{array}\right. \end{array}$$ (12)

where $\Gamma_{1}$ and $\Gamma_{2}$ denote the strategy sets of the subject vehicle (SV) and the following vehicle (LV) in the target lane, respectively. $\xi_{SV}^{*}$ is the optimal strategy of vehicle SV (the leader), and $\xi_{LV}^{*}(\xi_{SV})$ is the optimal response strategy of vehicle LV (the follower) to a given $\xi_{SV}$. *U*_*SV*_ and *U*_*LV*_ represent the decision payoffs of vehicles SV and LV, respectively.

The best response strategy set of vehicle LV corresponding to a given strategy $\xi_{SV}$ is defined as


$$\begin{array}{cc} A_{2}(\xi_{SV}) & =\text{arg}\max_{\xi_{LV}\in \Gamma_{2}}U_{LV}(\xi_{SV},\xi_{LV})\\ & =\{\gamma \in \Gamma_{2}:U_{LV}(\xi_{SV},\gamma )\geq U_{LV}(\xi_{SV},\xi_{LV}),\\ & \;\forall \xi_{LV}\in \Gamma_{2}\}. \end{array}$$ (13)

Accordingly, the optimal strategies of vehicles SV and LV can be obtained based on the above formulation.

### 3.3 Optimal lane-changing trajectory

In this paper, a cubic polynomial curve is adopted to generate the lane-changing trajectory of the vehicle. Such trajectories exhibit second-order smoothness [34], ensuring continuity of both position and velocity throughout the lane-changing process. Compared with higher-order polynomial trajectories, the cubic polynomial requires fewer parameters, thereby reducing the amount of information needed for trajectory generation. The cubic polynomial trajectory is formulated as follows:


$$\begin{array}{c} \left\{ \begin{array}{c} x(t)=a_{0}+a_{1}t+a_{2}t^{2}+a_{3}t^{3},\\ y(t)=b_{0}+b_{1}t+b_{2}t^{2}+b_{3}t^{3}, \end{array}\right. \end{array}$$ (12)

where *x*(*t*) and *y*(*t*) denote the longitudinal and lateral positions of the vehicle as functions of time *t*, respectively, and *a*_*i*_ and *b*_*i*_ (*i* = 0,1,2,3) are the coefficients of the cubic polynomial.

The cubic polynomial lane-changing trajectory of the autonomous vehicle and the corresponding trajectory curvature are illustrated in Fig 2.

To enable dynamic trajectory planning for vehicle lane-changing maneuvers, a moving coordinate system is adopted in this paper. Whenever trajectory planning is performed, a local coordinate frame is established with the current vehicle position taken as the origin. Furthermore, to simplify the trajectory generation process, it is assumed that the vehicle maintains a constant longitudinal speed throughout the lane-changing maneuver. Accordingly, the following boundary conditions are imposed:


$$\begin{array}{c} \left\{ \begin{array}{c} x(0)=0,\;x(T)=X,\;\dot{x}(0)=v\cos\theta_{j},\;\dot{x}(T)=v,\\ y(0)=0,\;y(T)=Y,\;\dot{y}(0)=v\sin\theta_{j},\;\dot{y}(T)=0, \end{array}\right. \end{array}$$ (13)

where *T* denotes the time required for the vehicle to complete the lane-changing maneuver from the current position, and $\theta_{j}$ represents the initial heading angle of the vehicle at the *j*-th trajectory planning step.


$$\begin{array}{c} \left\{ \begin{array}{c} x(t)=vt\cos\theta_{j}+\left(3X-vT-2vT\cos\theta_{j}\right)\;{\left(\frac{t}{T}\right)}^{2}+\left(vT\cos\theta_{j}+vT-2X\right)\;{\left(\frac{t}{T}\right)}^{3},\\ y(t)=vt\sin\theta_{j}+\left(3Y-2vT\sin\theta_{j}\right)\;{\left(\frac{t}{T}\right)}^{2}+\left(vT\sin\theta_{j}-2Y\right)\;{\left(\frac{t}{T}\right)}^{3}, \end{array}\right. \end{array}$$ (14)

where $\theta_{j}$ denotes the initial heading angle of the vehicle at the current time step, which can be obtained from the vehicle state, and *Y* represents the lateral distance from the local coordinate origin to the centerline of the target lane.

The lateral distance *Y* from the vehicle to the centerline of the target lane can be obtained from onboard sensors. The longitudinal distance *X* and the lane-changing duration *T* are treated as decision variables; therefore, the values of *X* and *T* jointly determine the final lane-changing trajectory.

**Fig 3:**
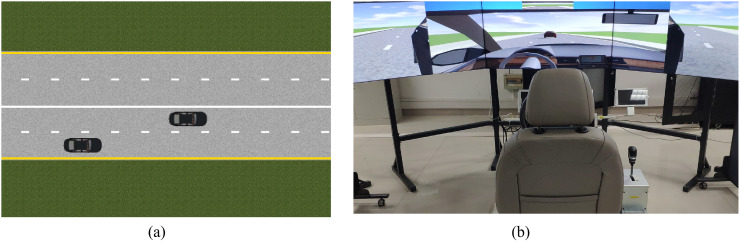
Simulation diagram.

In practical lane-changing scenarios, lane-changing efficiency and passenger comfort are two critical criteria for evaluating trajectory quality. However, these objectives are often conflicting and cannot be simultaneously optimized. As the vehicle approaches the lane-change prohibition region, the urgency to complete the maneuver increases, and priority should be given to minimizing the lane-changing duration rather than maximizing passenger comfort. Accordingly, the following cost function is defined:


$$\begin{array}{c} J=\chi^{\prime }v^{2}K_{\max}+(1-\chi^{\prime })T, \end{array}$$ (15)

where *J* denotes the cost function, $K_{\max}$ is the maximum curvature of the lane-changing trajectory, *T* represents the duration of the lane change, and *v* is the vehicle speed. The weighting coefficient $\chi^{\prime }$ reflects the urgency of the lane change and is defined as a function of the distance to the lane-change boundary:


$$\begin{array}{c} \chi^{\prime }=\frac{10\chi }{\sqrt{32\pi }}\exp\;\left(-\frac{(d_{x}-d_{2}{)}^{2}}{2\sigma_{1}^{2}}\right),\;d_{x}\in [0,d_{2}], \end{array}$$ (16)

where *d*_*x*_ denotes the longitudinal distance between the current vehicle position and position L2, *d*_2_ is a constant representing the boundary of the lane-change region, $\sigma_{1}$ is a positive constant, and χ is the initial value of the weighting coefficient. As illustrated in Fig 3, the curvature of the lane-changing trajectory reaches its maximum value at the completion of the maneuver. Accordingly, the maximum curvature of the lane-changing trajectory is defined as


$$\begin{array}{c} K_{\max}=\left|\frac{\dot{x}(T)\ddot{y}(T)-\ddot{x}(T)\dot{y}(T)}{{\left({\dot{x}}^{2}(T)+{\dot{y}}^{2}(T)\right)}^{3/2}}\right|, \end{array}$$ (17)

The solution of the cost function defined in (15) can be formulated as a nonlinear optimization problem. In this paper, the artificial fish swarm optimization (AFSO) algorithm is employed to solve the resulting optimization problem. By applying the AFSO algorithm, the optimal value of the cost function J^*^ can be obtained, along with the corresponding optimal lane-changing distance X^*^ and lane-changing duration T^*^.

### 3.4 Lane changing area calculation

Based on the previous analysis, the vehicle lane-changing region in the vicinity of the signalized intersection has been identified. To further characterize the vehicle’s operational state within this region, the lane-changing area is further subdivided according to the maximum lateral acceleration generated along the lane-changing trajectory.

By adopting a cubic polynomial lane-changing trajectory, the lateral acceleration of the vehicle can be expressed as


$$\begin{array}{c} a_{y}(t)=v^{2}\left|\frac{\dot{x}(t)\ddot{y}(t)-\ddot{x}(t)\dot{y}(t)}{{\left({\dot{x}}^{2}(t)+{\dot{y}}^{2}(t)\right)}^{3/2}}\right|, \end{array}$$ (18)

where *v* denotes the vehicle speed, and *x*(*t*) and *y*(*t*) represent the longitudinal and lateral positions of the vehicle at time *t*, respectively.

When the vehicle completes the lane-changing maneuver, the trajectory curvature reaches its maximum value; consequently, the maximum lateral acceleration is attained at this time instant and is given by


$$\begin{array}{c} a_{y}^{\max}=v^{2}\left|\frac{\dot{x}(T)\ddot{y}(T)-\ddot{x}(T)\dot{y}(T)}{{\left({\dot{x}}^{2}(T)+{\dot{y}}^{2}(T)\right)}^{3/2}}\right|, \end{array}$$ (19)

where *T* denotes the time required for the vehicle to complete the lane-changing maneuver. Considering the relationship between vehicle speed and displacement, the following expression can be further derived:


$$\begin{array}{c} v_{T}=\int_{0}^{X}\sqrt{1+{\left(\frac{\dot{y}(t)}{\dot{x}(t)}\right)}^{2}}\;dx, \end{array}$$ (20)

where *X* denotes the lane-changing distance of the vehicle.

To determine the length of *d*_2_ in Fig 1, the maximum lateral acceleration that can be tolerated by passengers without inducing vehicle rollover is denoted by $a_{\max}$. By jointly considering (19) and (20), the minimum feasible lane-changing distance can be obtained as $X_{\min}$, which corresponds to the length of *d*_2_. Accordingly, the minimum lane-changing duration is denoted as $T_{\min}$.

By jointly considering lane-changing efficiency and passenger comfort, the optimal lane-changing distance X^*^ is obtained using the artificial fish swarm optimization algorithm. As a result, the length of region *d*_1_ can be computed as


$$\begin{array}{c} d_{1}=X^{*}-d_{2}. \end{array}$$ (21)

The length of the guide lane *d*_3_ is assumed to be known a priori.

### 3.5 Prescribed performance control

To facilitate controller design under performance constraints, the constrained tracking error is transformed into an equivalent unconstrained form through a predefined error transformation function. Intuitively, this transformation maps the original error, which is required to evolve within a prescribed performance boundary, into a new variable defined over an unconstrained domain. As a result, the difficulty of directly handling state constraints is avoided, and standard control design techniques can be applied more conveniently.

Moreover, this transformation ensures that as long as the transformed error remains bounded, the original tracking error will automatically satisfy the predefined performance constraints, including both transient and steady-state requirements. This provides a systematic way to enforce safety-related bounds on tracking errors while maintaining analytical tractability. Compared with directly constrained control methods, the proposed transformation simplifies the control design process and enhances robustness, making it particularly suitable for safety-critical and highly interactive lane-changing scenarios.


$$\begin{array}{c} \left\{ \begin{array}{c} \dot{\beta }=-\frac{k_{1}+k_{2}}{mv_{x}}\beta +\left(\frac{ak_{1}-bk_{2}}{mv_{x}^{2}}-1\right)\omega -\frac{k_{1}}{mv_{x}}\delta_{f},\\ \dot{\omega }=\frac{ak_{1}-bk_{2}}{I_{z}}\beta +\frac{a^{2}k_{1}+b^{2}k_{2}}{I_{z}v_{x}}\omega -\frac{ak_{1}}{I_{z}}\delta_{f}, \end{array}\right. \end{array}$$ (22)

where *m* denotes the vehicle mass, $\delta_{f}$ is the front-wheel steering angle, *v*_*x*_ and *v*_*y*_ represent the longitudinal and lateral velocity components in the vehicle coordinate system, respectively, ω denotes the yaw rate, and *I*_*z*_ is the moment of inertia of the vehicle about the vertical axis. *a* and *b* denote the distances from the vehicle centroid to the front axle and rear axle, respectively, β represents the vehicle sideslip angle at the centroid, *k*_1_ and *k*_2_ are the cornering stiffness coefficients of the front and rear wheels, respectively.

In the trajectory tracking process of autonomous vehicles, it is necessary to regulate both the lateral tracking error and the heading angle error. Accordingly, the lane-changing trajectory tracking error model is defined as follows:


$$\begin{array}{c} \left\{ \begin{array}{c} {\dot{e}}_{y}=v_{x}\sin(e_{\theta })+v_{y}\cos(e_{\theta }),\\ e_{\theta }=\theta -\theta_{ref}, \end{array}\right. \end{array}$$ (23)

where *e*_*y*_ denotes the lateral tracking error, $e_{\theta }$ is the heading angle error, θ represents the actual heading angle of the vehicle, and $\theta_{ref}$ denotes the desired heading angle along the reference trajectory.

The core idea of prescribed performance control (PPC) is to confine the system tracking error within a predefined performance envelope, such that the tracking error converges to a prescribed residual set with bounded overshoot and guaranteed minimum convergence rate. To alleviate the complexity introduced by the performance constraints in controller design, an error transformation function is introduced to map the constrained nonlinear system into an unconstrained form:


$$\begin{array}{c} S(\varepsilon )=\left\{ \begin{array}{cc} \frac{e^{\varepsilon }-\delta e^{-\varepsilon }}{e^{\varepsilon }+e^{-\varepsilon }}, & e(t)\geq 0,\\ \frac{\delta e^{\varepsilon }-e^{-\varepsilon }}{e^{\varepsilon }+e^{-\varepsilon }}, & e(t)<0, \end{array}\right. \end{array}$$ (24)

where *e*(*t*) denotes the initial tracking error of the controlled system, ε is the transformed error variable, and δ is the overshoot suppression parameter.

By substituting the lateral tracking error and heading angle error into (24), the corresponding equivalent error dynamics can be obtained as follows:


$$\begin{array}{c} {}[\begin{array}{c} \varepsilon_{y}\\ \varepsilon_{\theta } \end{array}]=[\begin{array}{c} \frac{1}{2}\ln\;\left(\delta +\frac{e_{y}}{\eta }\right)-\frac{1}{2}\ln\;\left(1-\frac{e_{y}}{\eta }\right)\\ \frac{1}{2}\ln\;\left(\delta +\frac{e_{\theta }}{\eta }\right)-\frac{1}{2}\ln\;\left(1-\frac{e_{\theta }}{\eta }\right) \end{array}], \end{array}$$ (25)

Recognizing that regulating only the front-wheel steering angle is insufficient to simultaneously suppress both lateral deviation and heading angle error, a more comprehensive control strategy is adopted that jointly accounts for these two error components. Building upon this concept, this study further introduces a unified error metric that integrates the equivalent transformations of both heading angle error and lateral deviation, thereby enabling more effective and coordinated error regulation.


$$\begin{array}{c} e_{m}=x_{n}\varepsilon_{y}+x_{m}\sin(\varepsilon_{\theta }), \end{array}$$ (26)

where *e*_*m*_ denotes the comprehensive tracking error, and *x*_*n*_ and *x*_*m*_ are positive proportional coefficients corresponding to the lateral deviation and the heading angle error, respectively.

Assuming that the heading angle deviation remains sufficiently small during vehicle motion, the small-angle approximation can be applied, yielding


$$\begin{array}{c} e_{m}=x_{n}\varepsilon_{y}+x_{m}\varepsilon_{\theta }. \end{array}$$ (27)

Owing to its favorable characteristics, including fast dynamic response and strong robustness against external disturbances and model uncertainties, sliding mode control is adopted to design the trajectory tracking controller. The sliding surface is constructed as


$$\begin{array}{c} S=c_{1}e_{m}+c_{2}{\dot{e}}_{m}+c_{3}\int_{0}^{t}e_{m}(\tau )\;d\tau , \end{array}$$ (28)

where *S* denotes the sliding surface, and *c*_1_, *c*_2_, and *c*_3_ are positive design coefficients of the sliding surface. *e*_*m*_ represents the comprehensive transformed tracking error of the system.

In this paper, a general reaching law is adopted, which is expressed as


$$\begin{array}{c} \dot{S}=-\varepsilon \;sgn(S)-f(S), \end{array}$$ (29)

where ε is a positive constant, *f*(*S*) > 0, and S≠0.

To mitigate the chattering phenomenon inherent in sliding mode control, the saturation function is employed to replace the discontinuous sign function:


$$\begin{array}{c} sat(S)=\left\{ \begin{array}{cc} 1, & S>\Delta ,\\ S, & |S|\leq \Delta ,\\ -1, & S<-\Delta , \end{array}\right. \end{array}$$ (30)

where Δ denotes the boundary layer thickness. By combining the above formulations, a prescribed-performance-based sliding mode controller is obtained as


$$\begin{array}{c} \delta_{f}=\frac{c_{2}\omega_{1}+c_{2}\omega_{2}+c_{2}\omega_{4}+c_{1}{\dot{e}}_{m}+c_{3}e_{m}+\varepsilon \;sat(S)+\varepsilon_{2}S}{-c_{2}\omega_{3}}, \end{array}$$ (31)

$$\begin{array}{c} \omega_{1}=x_{n}\omega_{y}\left(\frac{k_{1}+k_{2}}{mv_{x}}{\dot{e}}_{y}-\frac{k_{1}+k_{2}}{m}e_{\theta }+\frac{ak_{1}-bk_{2}}{mv_{x}}{\dot{e}}_{\theta }+\frac{ak_{1}-bk_{2}}{m}\rho -\rho v_{x}^{2}\right), \end{array}$$ (32)

$$\begin{array}{c} \omega_{2}=x_{m}\omega_{\theta }\left(\frac{ak_{1}-bk_{2}}{I_{z}v_{x}}{\dot{e}}_{y}-\frac{ak_{1}-bk_{2}}{I_{z}}e_{\theta }+\frac{a^{2}k_{1}+b^{2}k_{2}}{I_{z}v_{x}}{\dot{e}}_{\theta }+\frac{a^{2}k_{1}+b^{2}k_{2}}{I_{z}}\rho -v_{x}\dot{\rho }\right), \end{array}$$ (33)

$$\begin{array}{c} \omega_{3}=-\left(\frac{x_{n}\omega_{y}k_{1}}{m}+\frac{x_{m}\omega_{\theta }ak_{1}}{I_{z}}\right), \end{array}$$ (34)

$$\begin{array}{cc} \omega_{4}= & \;x_{n}\left[{\dot{\omega }}_{y}\left({\dot{e}}_{y}-\frac{e_{y}\dot{\eta }}{\eta }\right)-\omega_{y}\left(\frac{{\dot{e}}_{y}\dot{\eta }}{\eta }+\frac{e_{y}(\ddot{\eta }\eta -{\dot{\eta }}^{2})}{\eta^{2}}\right)\right]\\ & +x_{m}\left[{\dot{\omega }}_{\theta }\left({\dot{e}}_{\theta }-\frac{e_{\theta }\dot{\eta }}{\eta }\right)-\omega_{\theta }\left(\frac{{\dot{e}}_{\theta }\dot{\eta }}{\eta }+\frac{e_{\theta }(\ddot{\eta }\eta -{\dot{\eta }}^{2})}{\eta^{2}}\right)\right], \end{array}$$ (35)

$$\begin{array}{c} \omega_{y}=\frac{1}{2}\left(\frac{1}{\eta \delta +e_{y}}-\frac{1}{e_{y}-\eta }\right), \end{array}$$ (36)

## 4 Results

To evaluate the effectiveness of the proposed lane-changing framework, a joint simulation platform integrating MATLAB/Simulink and a driver-in-the-loop (DiL) simulator is developed. MATLAB/Simulink is used to implement the lane-changing decision-making and trajectory tracking control algorithms, providing a flexible environment for algorithm development and real-time execution. The driving simulator enables human participants to operate vehicles through steering wheel and pedal inputs, allowing realistic interaction between human-driven vehicles and the autonomous vehicle.

The platform adopts a co-simulation architecture with real-time data exchange between modules. At each simulation step, environmental and traffic state information, including relative positions, velocities, lane information, and surrounding vehicle states, are transmitted to MATLAB/Simulink through a synchronous communication interface. Based on these inputs, the decision-making and control modules compute the optimal strategy and corresponding control commands (e.g., steering angle and acceleration), which are used to update vehicle states in real time. This closed-loop interaction is executed under a fixed time-step synchronization mechanism, ensuring consistency among perception, decision-making, and control processes. In addition, the human driver’s inputs from the simulator are incorporated into the traffic flow in real time, enabling bidirectional interaction between the autonomous vehicle and human-driven vehicles.

In the simulation environment, an Audi A8 vehicle model is selected to represent the autonomous vehicle. The autonomous vehicle performs lane-changing decision-making and trajectory tracking control based on real-time environmental feedback. The following vehicle in the target lane is controlled by human participants via the driving simulator, forming a mixed traffic scenario that captures realistic interaction dynamics between autonomous and human-driven vehicles. It should be noted that this study focuses on interaction-aware lane-changing behavior, rather than driver takeover or shared control.

A preliminary driver-in-the-loop experiment involving three human participants with different driving tendencies (e.g., conservative and aggressive styles) was conducted to investigate interaction patterns in mandatory lane-changing scenarios. Each participant performed multiple trials under identical experimental conditions to improve the consistency and reliability of the observations. The duration of each lane-changing maneuver was approximately 5–8 seconds, corresponding to the critical interaction phase of the maneuver.

Basic participant information, such as driving experience, was recorded for reference. All participants were informed of the experimental procedures and provided written informed consent prior to participation. The collected data were anonymized and analyzed using a qualitative and descriptive approach, providing preliminary insights into interaction behaviors under different driving styles.

Remark 1: It should be noted that the driver-in-the-loop experiment in this study involves a limited number of participants. Therefore, the findings are intended to provide preliminary qualitative insights into interaction behaviors rather than statistically generalizable conclusions. Future work will include a larger and more diverse participant pool to further validate the proposed framework.

Remark 2: This study was reviewed and approved by the Academic Ethics Committee of Taiyuan University of Science and Technology. All participants provided written informed consent prior to participation in the study.

**Fig 4:**
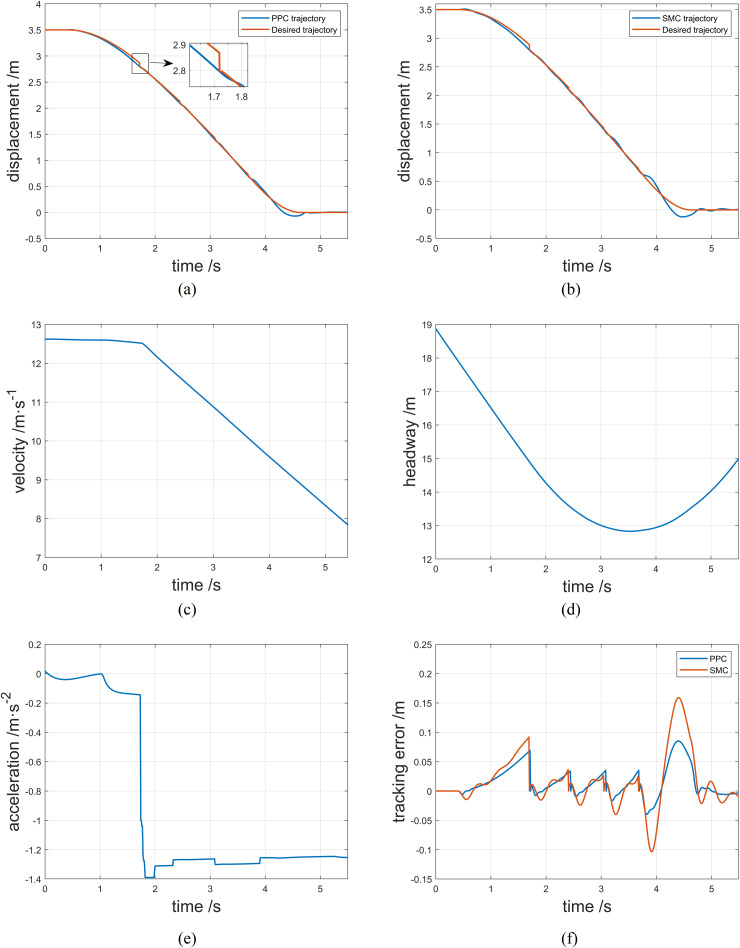
Condition I experimental diagram: **(a)** SV lateral trajectory controlled by PPC; **(b)** SV lateral trajectory controlled by SMC; **(c)** LV speed; **(d)** vehicle spacing between SV and LV; **(e)** LV acceleration; **(f)** trajectory tracking lateral error.

**Fig 5:**
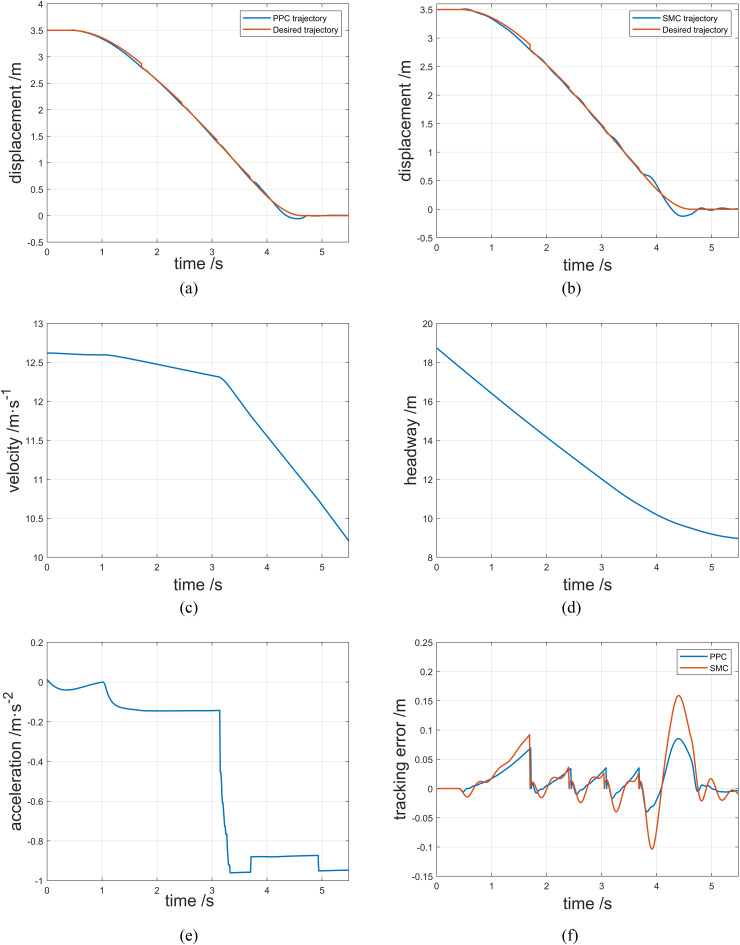
Condition II experimental diagram: **(a)** SV lateral trajectory controlled by PPC; **(b)** SV lateral trajectory controlled by SMC; **(c)** LV speed; **(d)** vehicle spacing between SV and LV; **(e)** LV acceleration; **(f)** trajectory tracking lateral error.

The results presented in Figs 4 and 5 illustrate the interaction patterns arising from relatively cautious driving styles, as exhibited by Driver 1 and Driver 2, and their influence on the lane-changing behavior of the subject vehicle (SV). Upon detecting the SV’s lane-change intention, both drivers adopt conservative strategies by decelerating to yield, forming a cooperative interaction pattern. This behavior effectively enlarges the available gap and reduces conflict intensity, thereby facilitating the successful execution of the lane-change maneuver. Although variations exist in the timing and magnitude of deceleration, the overall interaction consistently leads to a favorable outcome.

The corresponding lateral trajectory error profiles reveal multiple instances of trajectory re-planning during the lane-change process, indicating that the SV continuously adjusts its motion in response to surrounding vehicle behaviors. These fluctuations reflect the dynamic coupling between decision-making and control under interactive conditions. Importantly, such adaptive trajectory adjustments mitigate potential collision risks by compensating for disturbances induced by time-varying interactions, highlighting the robustness of the proposed framework in cooperative scenarios.

Furthermore, a comparative evaluation of trajectory tracking performance demonstrates the superiority of the proposed Preset Performance Control (PPC) approach over conventional Sliding Mode Control (SMC). Specifically, PPC achieves reduced lateral tracking errors and faster convergence rates, particularly during transient phases, which are critical for safety in lane-changing scenarios. This improved tracking performance enhances the system’s ability to respond promptly to interaction-induced disturbances, thereby lowering collision risk and ensuring smoother maneuver execution.

**Fig 6:**
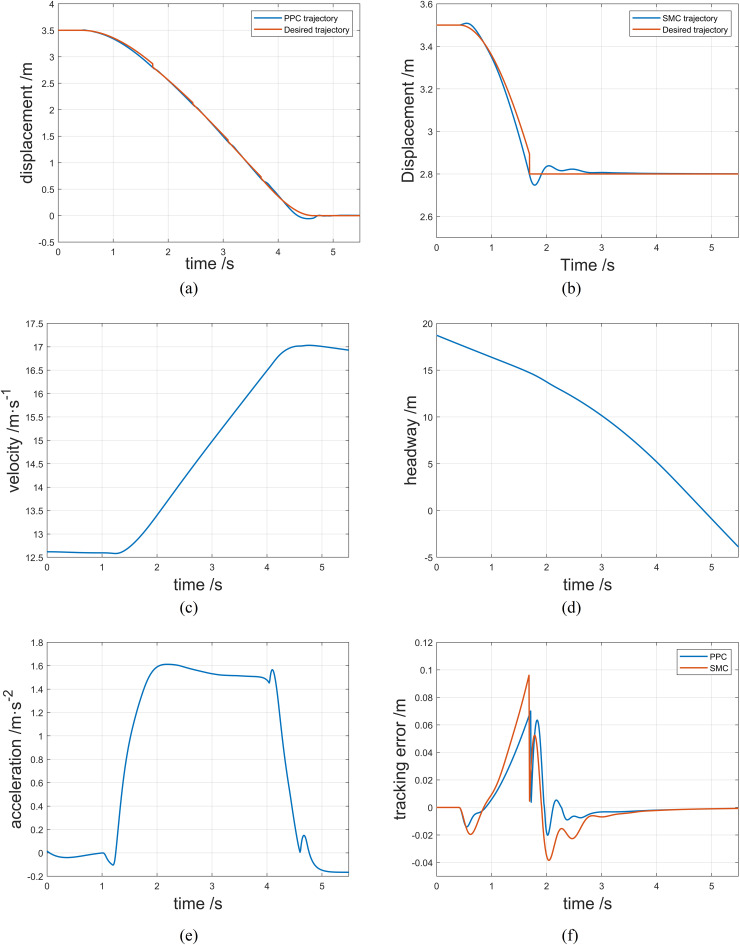
Condition III experimental diagram: **(a)** SV lateral trajectory controlled by PPC; **(b)** SV lateral trajectory controlled by SMC; **(c)** LV speed; **(d)** vehicle spacing between SV and LV; **(e)** LV acceleration; **(f)** trajectory tracking lateral error.

In contrast, the results in Fig 6 reveal a markedly different interaction pattern associated with the aggressive driving behavior of Driver 3. Upon perceiving the SV’s lane-change intention, Driver 3 adopts a competitive strategy by accelerating sharply (approximately at 1.2 s), which rapidly decreases the inter-vehicle distance and intensifies the interaction conflict. This aggressive response transforms the interaction from cooperative to competitive, significantly increasing risk levels.

In response to the elevated collision risk, the SV dynamically revises its decision and aborts the lane-change maneuver at around 1.8 s, maintaining its current lane. This outcome highlights the effectiveness of the proposed framework in identifying unsafe interaction patterns and triggering timely decision adjustments. It also demonstrates the framework’s capability to balance safety and efficiency under adverse conditions through real-time interaction-aware decision-making.

These findings underscore the critical impact of heterogeneous driving styles on lane-changing outcomes. Cooperative behaviors tend to reduce interaction intensity and promote maneuver feasibility, whereas aggressive behaviors amplify conflicts and may invalidate previously feasible lane-changing plans, necessitating adaptive strategy revisions. Overall, the results confirm that the proposed game-based framework enables the SV to dynamically adapt its decision-making strategy by incorporating real-time interaction feedback and environmental information. In addition, the proposed trajectory tracking control method enhances safety by reinitializing tracking errors during trajectory re-planning and enforcing constrained transient responses. Compared with conventional SMC, the proposed approach exhibits improved transient and steady-state performance, providing a more reliable and safety-oriented solution for autonomous vehicle lane-changing in complex traffic environments.

## 5 Conclusion

This paper investigates vehicle lane-changing behavior in mandatory lane-changing scenarios, with a particular focus on traffic environments in the vicinity of signalized intersections. To capture the unique characteristics of such scenarios, the near-signal road region is systematically partitioned into a lane-change decision area and a lane-change prohibition area based on the admissible lateral acceleration constraints of lane-changing maneuvers. By explicitly incorporating lane-changing motivation into the formulation, the payoff function of the lane-changing vehicle is refined, and both the weighting coefficients and the optimal lane-changing distance are adaptively adjusted according to the vehicle’s spatial position. Building upon these developments, an integrated lane-changing decision and control framework tailored to near-signal traffic environments is established.

The experimental results demonstrate that the proposed framework is capable of interacting effectively with drivers exhibiting heterogeneous driving styles, while dynamically adapting driving strategies in response to real-time interaction feedback. In addition, the proposed control approach enables accurate and robust trajectory tracking under dynamically changing conditions. These findings provide evidence that the proposed method can enhance interaction awareness and operational safety in complex mandatory lane-changing scenarios.

Despite these promising findings, it should be noted that the current study is based on a limited number of human participants and relies primarily on simulation-based evaluation. Future work will focus on extending the experimental scale, incorporating more comprehensive statistical analyses, and validating the proposed framework in more diverse and realistic traffic environments, including real-world testing.
